# Thyroid hormones enhance growth and counteract apoptosis in human tenocytes isolated from rotator cuff tendons

**DOI:** 10.1038/cddis.2013.229

**Published:** 2013-07-04

**Authors:** F Oliva, A C Berardi, S Misiti, C V Falzacappa, A Iacone, N Maffulli

**Affiliations:** 1 Department of Orthopaedics and Traumatology, University of Rome ‘‘Tor Vergata'' School of Medicine, Viale Oxford 81, Rome, Italy; 2 Laboratory of Stem Cells, Department of Transfusion Medicine, Spirito Santo Hospital, Pescara, Italy; 3 Department of Experimental Medicine, Endocrinology, Sapienza University of Rome, Rome, Italy; 4 Faculty of Medicine and Surgery, Department of Musculoskeletal Medicine and Surgery, University of Salerno, Salerno, Italy; 5 Newham University Hospital, London, UK; 6 Centre for Sports and Exercise Medicine – Queen Mary, University of London, Barts, UK

*Dear Editor*,

The relationship between thyroid disorders and shoulder pain has been suspected since the late 1920s.^1^ More recently, such association has been more formally hypothesized,^2^ and thyroid diseases have been linked to idiopathic tendinopathies.^3, 4^

The essential role of thyroid hormones (THs), T_3_ (triiodothyronine) and T_4_ (thyroxine), in the development and metabolism of many tissues and organs, both in early and adult life,^5^ is mediated mainly through T_3_, which regulates gene expression by binding to the TH receptors (TRs)-*α* and -*β*.

Although TRs seem to be ubiquitous, their presence on tendons has not been previously investigated. We therefore evaluated the expression pattern of TR isoforms in three groups of patients: one with rotator cuff tendon tears and thyroid diseases, one with rotator cuff tendon tears without thyroid diseases, and one with healthy rotator cuff tendons. The TR*α* and TR*β* protein expression level was characterized by western blot analysis (Figure 1a and Supplementary Figure S1a). All healthy and pathologic rotator cuff tendons analyzed expressed high levels of TR*α*/*β* nuclear receptor isoforms, indicating that TR*α*/*β* expression pattern is not influenced by thyroid diseases.

THs regulate cell proliferation. To further investigate the role played by TRs, we designed *in vitro* condition that may allow THs to induce proliferation of tenocytes. To this end, tenocytes were isolated from human tendon tissues obtained from five healthy subjects,^6^ and cultured for 72 h with or without THs. As expected, both T_3_ and T_4_ induced cell growth, most effectively at 10^−7^ M. The higher increase was obtained by 72 h of hormone treatment, being 19% for T_3_ and 10% for T_4_. Tenocytes grew with a doubling time of approximately 49 h. The addition of the THs in the culture medium led to stimulation of cell growth with a reduction of the doubling time. In particular, T_3_ induced a reduction in doubling time of 27% (36 h) and T_4_ of 19% (40 h; Figure 1b), with the 10^−7^ M dose.

T_3_ and T_4_ play an antiapoptotic action.^7, 8^ Hence, to verify whether they counteracted apoptosis in isolated tenocytes, cells were plated and serum deprivation was performed for 48 h to induce apoptosis. Concurrently, tenocytes were exposed or not (control) to T_3_ or T_4_. Staining cells simultaneously with Annexin V and the non-vital dye propidium iodide (PI) allows to identify live (Annexin V^−^ PI^−^), apoptotic (Annexin V^+^ PI^−^) and necrotic cells (Annexin V^+^PI^+^). T_3_ and T_4_ caused an increase in vital cells (83, 81 *versus* 62%) and a reduction of apoptotic (5.6, 7.1 *versus*18.6%) cells after 48 h compared with the control cells (Supplementary Figure S1b).

These results demonstrate that the TR*α*/*β* nuclear receptor isoforms are present in healthy and pathologic rotator cuff tendons. THs enhance, *in vitro*, tenocyte growth, and counteract apoptosis in healthy tenocytes isolated from tendon in a dose- and time-dependent manner. Taken together, these results reinforce the concept of a physiological action of THs in the homeostasis of tendons. Much research remains to be performed to clarify the exact role of THs in tendon tissues and their implications in tendon ruptures, tendinopathies and tendon healing. If this association is confirmed, assessment and treatment of patients with tendon conditions may have to be revisited.

## Figures and Tables

**Figure 1:**
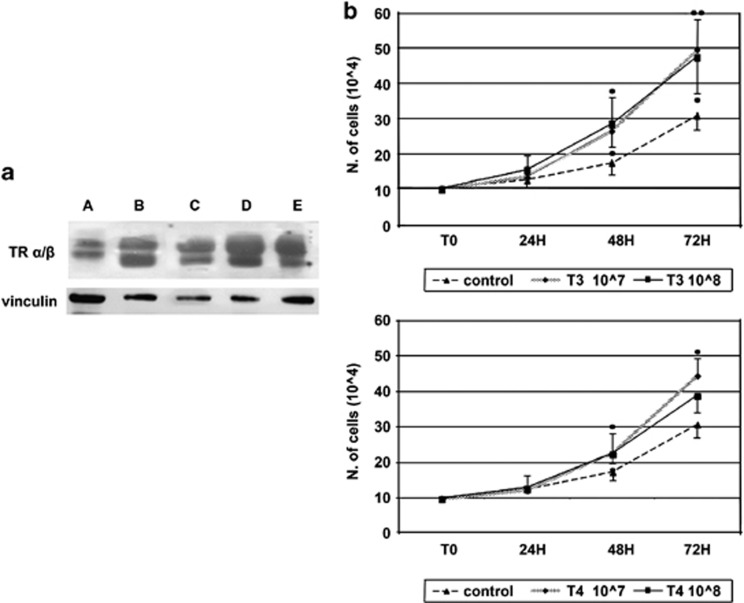
(**a**) Western blot analysis of TR*α*/*β* isoforms. A indicates patients with healthy rotator cuff tendons; B–C indicate patients with rotator cuff tears without thyroid disease, D–E represent patients with rotator cuff tears and thyroid disease. The polyclonal antibodies against TRs *α*/*β* recognize two specific bands at 47 and 55 kDa, respectively. At least three different experiments were performed, and a representative one is shown here. (**b**) Cell growth: tenocyte isolated from healthy tendon were cultured and exposed to different THs concentrations. The graphic shows the effect of T_3_ and T_4_ treatment on cell growth determined by counting trypan blue negative cells; Y axis: cell number, X axis: hours of THs treatment. All the data are presented as mean±SD, and are the results from five individual experiments. A comparison of the individual treatment was conducted by using one-way ANOVA followed by Turkey *post hoc* test. Statistical significance in comparison with the corresponding control values indicated by *P<0.05 *versus* control; **P<0.01 *versus* control
