# Supplementary figures and images for: Thyroid hormones enhance growth and counteract apoptosis in human tenocytes isolated from rotator cuff tendons
Source: Cell Death Dis. 2013 Jul 4;4(7):e705–. doi: 10.1038/cddis.2013.229 (PMC3730403; doi:10.1038/cddis.2013.229)

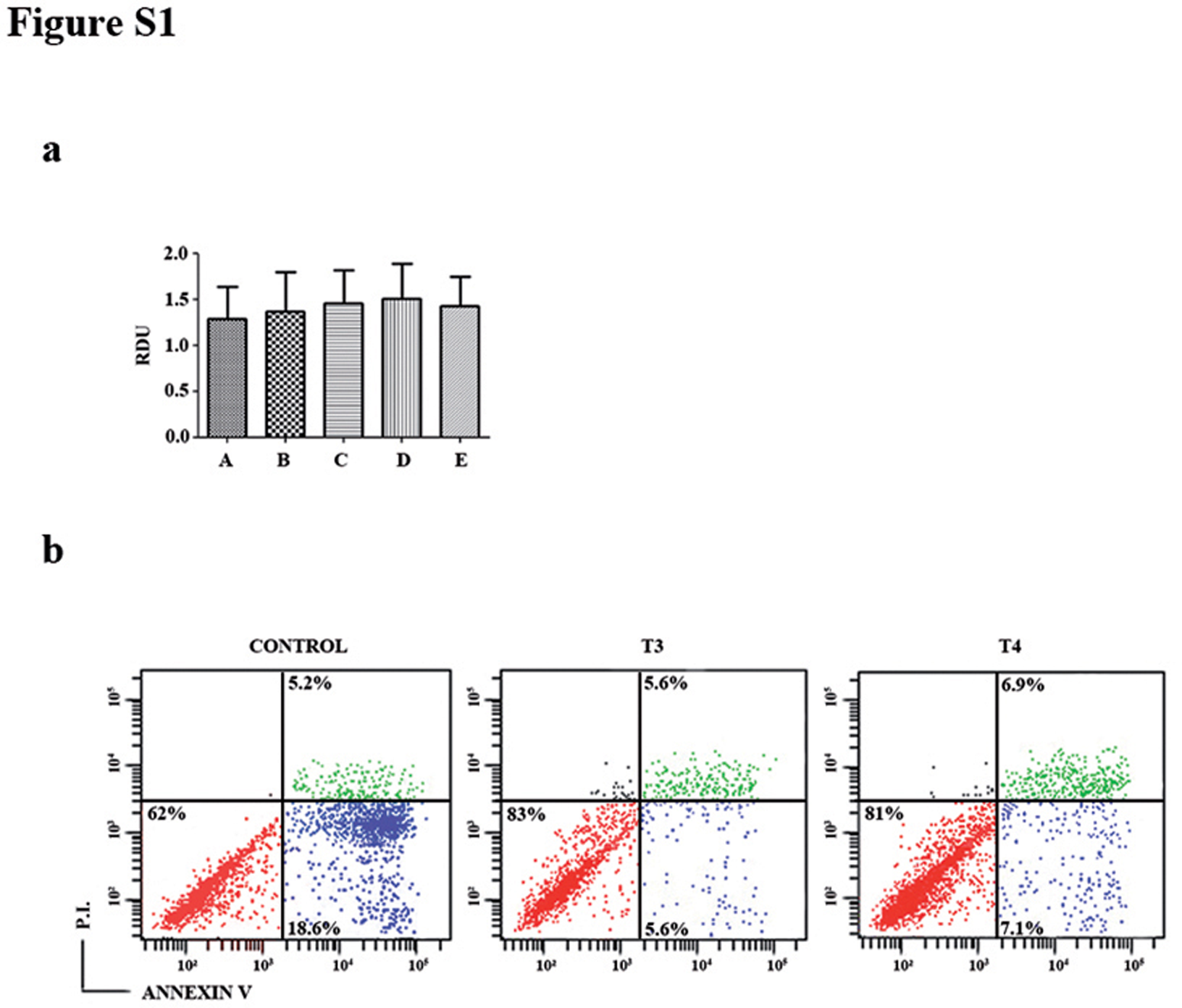

Supplement: Supplementary Figure [file cddis2013229x1.tif]
