# Supplementary material for: Thyroid hormones enhance growth and counteract apoptosis in human tenocytes isolated from rotator cuff tendons
Source: Cell Death Dis. 2013 Jul 4;4(7):e705–. doi: 10.1038/cddis.2013.229 (PMC3730403; doi:10.1038/cddis.2013.229)
Supplement: Supplementary Figure Legend [file cddis2013229x2.doc]

**Supplemental Legend: Figure S1. a.** *Western blot analysis of TRα/β isoforms*. A: patients with healthy rotator cuff tendons; B-C: patients with rotator cuff tears without thyroid disease, D-E: patients with rotator cuff tears and thyroid disease. Densitometric absorbance values from three separate experiments were averaged (± SD), after they had been normalized to Vinculin for equal loading. Data relative to each protein are presented in the histogram of the Western Blot as Relative Densitometric Units (y axis). **b.** *Determination of apoptosis by Annexin V assay.* At 48h after culture, the tenocytes in serum deprived medium, double staining with Annexin V/PI, apoptotic cell population were evaluated by flow cytometry. Results from one representative experiment of three independent experiments with similar finding are shown. Annexin V (staining is on the X-axis, and PI incorporation is on the Y-axis). Annexin V^-^PI^−^ cells were considered as vital (in red), Annexin V^+^PI^−^ cells were considered as apoptotic (in blue), Annexin V^+^PI^+^ cells as necrotic (in green). The percentage of control cells (untreated) and treated (T_3_ or T_4_) and of vital cells where 62%, 83%, 81% respectively; apoptotic cells 18.6%, 5.6%, 7.1% respectively. The results are expressed as percentage of total cells. Results are the mean of three independent experiments.
